# Commentary: Innovative machine learning-based prediction of early airway hyperresponsiveness using baseline pulmonary function parameters

**DOI:** 10.3389/fmed.2026.1775063

**Published:** 2026-03-23

**Authors:** Kazim Okan Dolu

**Affiliations:** Department of Pediatric Allergy and Immunology, Kanuni Sultan Süleyman Training and Research Hospital, Istanbul, Türkiye

**Keywords:** airway hyperresponsiveness, asthma, machine learning, predictive model, pulmonary function parameters

## Introduction

Yang et al. developed a machine learning-based nomogram to predict airway hyperresponsiveness (AHR) among patients with suspected asthma using baseline pulmonary function parameters (1). While the clinical aim is relevant, I identified several internal inconsistencies in statistical reporting that currently limit interpretability and reproducibility of the model and warrant formal correction.

## Analysis

### Coefficient-confidence interval inconsistency (Table 2, Model C)

Table 2 reports the Model C coefficient for MMEF75-25%pred as −0.31 with a 95% confidence interval (CI) of (−0.06, −0.006) (1). By statistical definition, a 95% CI must contain its point estimate; here the reported interval does not include the coefficient. This appears to be a reporting error (e.g., transcription of the coefficient and/or CI) and directly affects the nomogram because coefficient weights determine individual risk calculations. I request confirmation and correction of the exact coefficient and its 95% CI for this predictor, alongside any downstream updates required for the nomogram and Figure 4.

### AUC and 95% CI inconsistency and ambiguity (results)

The Results section reports an AUC of 0.790 for the training cohort while also presenting a 95% CI of (0.724–0.760) (1). This interval does not include the stated AUC value, and it is presented adjacent to the ‘optimal cut-off' value (0.354), creating ambiguity as to whether the interval pertains to AUC or another quantity. Similar ambiguity appears for the validation cohort (AUC = 0.756; CI: 0.600–0.814) (1). I ask the authors to provide the correct AUC estimates and their corresponding 95% CIs for both cohorts, and to clearly label which CI belongs to which metric. Figure 5 should also be updated to reflect corrected values.

### Missing methodological detail affecting internal validation (imputation timing)

The Methods describe random splitting into training/validation groups and the use of multiple imputation by chained equations (MICE) for missing values, with predictors including ‘all variables in the analysis' (1). However, the manuscript does not explicitly state whether imputation was performed strictly within the training set (and then applied to the validation set) or on the combined dataset prior to splitting. Because pre-split imputation can introduce information leakage that compromises the independence of internal validation, I request explicit clarification of the exact sequence (i.e., split → impute within training only → apply to validation, or an alternative approach). If a different sequence was used, the authors should discuss potential implications for the reported validation performance (AUC = 0.756).

## Discussion

Given the statistical inconsistencies documented above—particularly the coefficient-CI mismatch and the AUC-CI reporting ambiguity—I respectfully request that the journal and authors issue a formal Correction/Addendum to: (i) correct the Table 2 coefficient and CI for MMEF75-25%pred in Model C, (ii) provide accurate AUC values and correctly labeled 95% CIs for both cohorts in the text and Figure 5, and (iii) clarify imputation timing relative to data splitting. These corrections are essential to ensure accurate interpretation and clinical implementation of the proposed model. To facilitate reproducibility, I also encourage sharing analysis code and, where feasible, de-identified data via a public repository.

## References

[1] Yang H Zhao X Chen Z Yang L Zhao G Xu C . Innovative machine learning-based prediction of early airway hyperresponsiveness using baseline pulmonary function parameters. Front Med. (2025) 12:1611683. doi: 10.3389/fmed.2025.1611683 40832096 PMC12358348

